# Food and Beverage Consumption and Melanoma Risk: A Population-Based Case-Control Study in Northern Italy

**DOI:** 10.3390/nu11092206

**Published:** 2019-09-12

**Authors:** Carlotta Malagoli, Marcella Malavolti, Francesca Farnetani, Caterina Longo, Tommaso Filippini, Giovanni Pellacani, Marco Vinceti

**Affiliations:** 1 Environmental, Genetic and Nutritional Epidemiology Research Center (CREAGEN), Section of Public Health–Department of Biomedical, Metabolic and Neural Sciences, University of Modena and Reggio Emilia, Via G. Campi 287, 41125 Modena, Italy; carlotta.malagoli@unimore.it (C.M.); marcella.malavolti@unimore.it (M.M.); tommaso.filippini@unimore.it (T.F.); 2 Dermatologic Unit, University of Modena and Reggio Emilia, Via Del Pozzo 71, 41124 Modena, Italy; francesca.farnetani@unimore.it (F.F.); caterina.longo@unimore.it (C.L.); giovanni.pellacani@unimore.it (G.P.); 3 Department of Epidemiology, Boston University School of Public Health, Boston, MA 02118, USA

**Keywords:** diet, food, melanoma, risk, case-control study, epidemiology

## Abstract

It has been suggested that diet may influence the risk of melanoma, but few studies are available on this topic. We assessed the relation between food consumption and the risk of cutaneous melanoma in a Northern Italy population. We carried out a population-based case-control study involving 380 cases of melanoma and 719 age- and sex-matched controls. Dietary habits were established through a self-administered semi-quantitative food frequency questionnaire. We computed the odds ratios (ORs) of melanoma and the corresponding 95% confidence intervals (CIs) according to tertiles of daily intake of each food item, using multiple logistic regression models adjusted for major confounding factors. We observed an indication of a positive association between melanoma risk and consumption of cereals and cereal products (OR = 1.32; 95% CI 0.89–1.96, higher vs. lowest tertile), sweets (OR = 1.22; 95% CI 0.84–1.76), chocolate, candy bars. etc., (OR = 1.51; 95% CI 1.09–2.09) and cabbages (OR = 1.51; 95% CI 1.09–2.09). Conversely, an inverse association with disease risk was found for the intake of legumes (OR = 0.77; 95% CI 0.52–1.13), olive oil (OR = 0.77; 95% CI 0.51–1.16), eggs (OR = 0.58; 95% CI 0.41–0.82), and onion and garlic (OR = 0.80; 95% CI 0.52–1.14). No relationship was observed with beverage consumption. Our results suggest potentially adverse effects on melanoma risk of foods characterized by high contents of refined flours and sugars, while suggesting a protective role for eggs and two key components of the Mediterranean diet, legumes and olive oil. These associations warrant further investigation and, if confirmed, they might have important public health implications for the reduction of melanoma incidence through dietary modification.

## 1. Introduction

Cutaneous malignant melanoma is the most serious type of skin cancer [1]. In Europe, its incidence has recently increased more rapidly than any other cancer, with an annual incidence of 13.5 new cases per 100,000 inhabitants in Northern and Western Europe [2]. The few established risk factors for melanoma include unconscionable and intermittent sun exposure, tanning bed use, severe sunburns, as well as phenotypical characteristics such as light skin, light eye color, red/blonde hair and low tanning ability [3].

In recent years, attention has been paid to the role of dietary habits and nutrient intake in melanoma risk [4,5,6,7,8,9]. Several epidemiological investigations have shown a trend towards reduced melanoma risk associated with a higher intake of beta-carotene and vitamins A, C, D, E [10,11,12]. In addition, healthy dietary patterns such as the Mediterranean diet, characterized by high consumption of vegetables, fruit, olive oil, moderate consumption of fish and wine, and low dairy and meat consumption, have been suggested to exert a protective effect [7,13]. In particular, vegetables, fruit, fish [14] and caffeine [15] showed a protective effect, while alcohol [16,17] as well as a diet rich in sugars/carbohydrates [18] and characterized by high glycemic load [6] may play detrimental roles.

In this study, we aimed at assessing the relation between consumption of principal food items and melanoma risk in a Northern Italy population, generally characterized by Mediterranean-like eating habits [7] and in which we have already investigated the effect of a number of dietary constituents on disease risk.

## 2. Methods

Details of our population-based case-control study on dietary risk factors of melanoma in the population of five provinces of the Emilia Romagna region in Northern Italy (more than 3 m residents), have been provided elsewhere [6,7]. Briefly, in the years 2005–2006, we attempted to recruit all patients with newly diagnosed melanoma residing in the provinces of Bologna, Ferrara, Modena, Parma and Reggio Emilia and attending the local dermatological clinics. Inclusion criteria were a histologically-confirmed diagnosis of melanoma without clinical evidence of metastasis. Overall, 572 eligible patients were contacted by their dermatologists to participate in the study, and 394 (69%) agreed to participate and completed the study questionnaires (see detail below). Six referents matched to each case for sex, year of birth (±5 years) and province of residence were randomly selected from the database of Emilia-Romagna within the National Health Service directory. An envelope containing the study questionnaires and a pre-paid return envelope was mailed to 2,825 potential controls and 747 (26%) agreed to participate in the study and returned the questionnaires. Fourteen cases and 28 controls were excluded from the subsequent analysis due to data incompleteness or extreme values derived from the food-frequency questionnaire (ratio of total energy intake and sex-specific basal metabolic rate calculated through Harris-Benedict formula <0.5th percentile or >99.5th percentile) [19]. Informed consent was obtained from all individual participants included in the study. This study was conducted according to the guidelines laid down in the Declaration of Helsinki. Written informed consent was obtained from all subjects/patients.

### 2.1. Dietary Assessment

Dietary habits during the year prior to enrolment were established using a self-administered semi-quantitative food frequency questionnaire, designed and validated to capture eating behaviors in Italy, and specifically developed as part of the European Prospective Investigation into Cancer and Nutrition (EPIC) study for the Northern Italy population [20,21]. Participants were asked to respond to 248 questions about 188 different food items, in order to assess frequency and quantity of daily consumption for each food item. Foods and beverages were categorized into major food groups and sub-groups based on the common EPIC-SOFT classification, as previously reported [22,23]. In addition, total energy and detailed nutrient intake were also calculated for each participant on the basis of the Italian food composition tables [21,24].

### 2.2. Additional Variables

Each participant provided information on place and date of birth, province of residence, educational level (≤5, 6–8, 9–13 or >13 years), marital status (married, unmarried/single, divorced or widowed), weight and height, phenotypic characteristics (eye, hair and skin color), sunburn history (never, first before or after 18 years of age) and skin sun reaction (speed of tan and tendency to burn). In detail, eye color was classified as follows: light (blue/green), light brown and dark (brown/black); hair color was classified as blond, red, light brown or dark brown/black at 20 years; skin color was classified as white, light brown, brown/olive or dark brown/ebony; skin sun reaction was classified as high tendency to burn and never tan, high tendency to burn and moderate tan, moderate tendency to burn and gradual tan, no tendency to burn and golden tan, no tendency to burn and intense tan [10,11]. Based on these categories, each subject was assigned to a phototype using the Fitzpatrick phototyping scale. We computed body mass index (BMI) as weight/height^2^ (kg/m^2^) and we categorized subjects as underweight (≤19 kg/m^2^), normal (20–24 kg/m^2^), overweight (25–29 kg/m^2^) and obese (≥30 kg/m^2^).

For each study participant, we computed the score for the a priori defined diet quality index, the Greek variant of Mediterranean diet Index (GMI). GMI scores (ranking 0 to 9) were formulated as Mediterranean diet scales [25], taking into account extensive evidence supporting the notion of beneficial effects of the Mediterranean diet in preventing cancer [26], including melanoma [7]. Additionally, we included the following factors as possible confounders: the daily average values for dietary glycemic load (GL) and dietary glycemic index (GI) [6].

### 2.3. Statistical Analysis

We adjusted the food item daily intake for total energy according to the Willet regression-residual method [27], and we categorized it into tertiles based on the distribution of residuals in the control group. Subjects who did not consume a food item were placed in the lowest tertile. We used multivariate conditional logistic regression models to estimate odds ratios (ORs) and 95% confidence intervals (CIs) of melanoma for tertiles of intake, with the lowest tertile as a reference. Along with matching variables (sex, age and province of residence), we included the subsequent variables as possible confounders: phototype (four categories), sunburn history (three categories), education (four categories), BMI (four categories), and non-alcohol energy intake (continuous). Additional dietary factors shown to affect melanoma risk in the study population are included as additional confounding factors, namely vitamin C (continuous), vitamin D (continuous), GMI score (continuous) and dietary GL value (continuous) [28]. Tests of linear trend were performed according to 1-g or 10-g increments of daily intake. We also carried out stratified analysis by sex, age, phototype and quality of diet as potential effect modifiers. Finally, we modeled the relation between food intake and melanoma risk using restricted cubic splines, computed with the ‘mkspline’ and ‘xblc’ routines of the Stata-15.1 statistical package (Stata Corp., College Station, TX, USA, 2017) [29], by selecting the optimal number of knots according to Akaike’s information criterion (AIC) and using the knot placement method recommended by Harrell [30].

## 3. Results

A total of 380 patients (175 males and 205 females, mean age 58 ± 16 and 53 ± 15 years, respectively) and 719 referents matched for age, sex and province of residence were included in the analysis. Baseline characteristics, and median with interquartile range of daily intake are shown in Table 1; Table 2 for each food item, within case and control groups.

**Figure 1:**
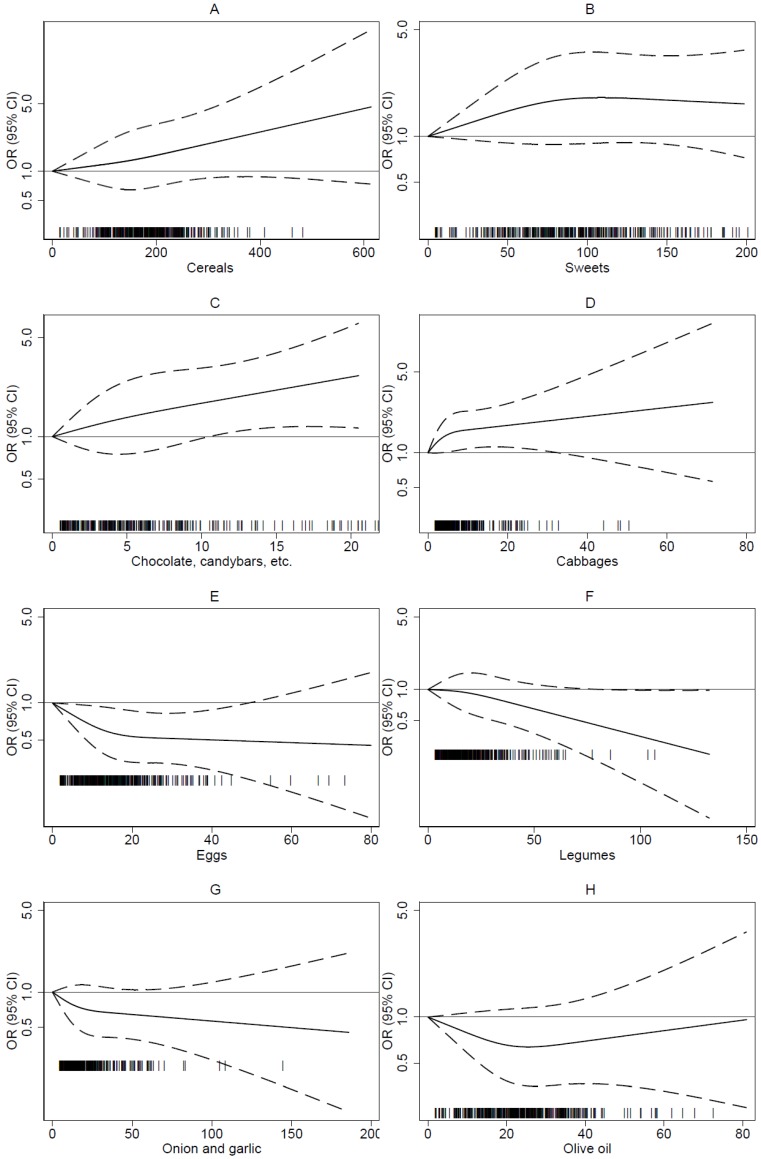
Spline regression analysis of the odds of being a case according to food item consumption (g/day), adjusting for phototype, sunburn history, education, body mass index, non-alcohol energy, vitamin C and vitamin D intake, Greek Mediterranean index and glycemic index; dotted lines, 95% confidence limits; reference line at 1.0. (**A**) Cereals; (**B**) Sweets; (**C**) Chocolate, candy bars, etc.; (**D**) Cabbages; (**E**) Eggs; (**F**) Legumes; (**G**) Onion and garlic; (**H**) Olive oil.

Table 3 provides ORs and 95% CIs for developing melanoma according to tertiles of daily intake for each food item, and controlling for potential confounders. In the fully adjusted model, we observed a direct association for higher consumers of the subsequent food categories: ‘Cereals and cereal products’ (OR = 1.32, 95% CI 0.89–1.96 higher vs. lowest tertile and OR = 1.02, 95% CI 1.00–1.05 linear trend), ‘Sweets’ (OR = 1.22, 95% CI 0.84–1.76 higher vs. lowest tertile and OR = 1.01, 95% CI 0.98–1.04 linear trend), particularly ‘Chocolate, candy bars etc.’ (OR = 1.51, 95% CI 1.09–2.09 higher vs. lowest tertile and OR = 1.05, 95% CI 0.88–1.26 linear trend) and ‘Cabbages’ (OR = 1.51, 95% CI 1.09–2.09 higher vs. lowest tertile and OR = 1.02, 95% CI 1.00–1.04 linear trend). Conversely, an inverse correlation with melanoma risk was observed for high consumption of the following food items: ‘Legumes’ (OR = 0.77, 95% CI 0.52–1.13 higher vs. lowest tertile and OR = 0.91, 95% CI 0.82–1.00 linear trend), ‘Olive oil’ (OR = 0.77, 95% CI 0.51–1.16 higher vs. lowest tertile and OR = 0.95, 95% CI 0.81–1.10 linear trend), ‘Eggs’ (OR = 0.58, 95% CI 0.41–0.82 higher vs. lowest tertile and OR = 0.98, 95% CI 0.73–0.97 linear trend), and ‘Onion and garlic’ (OR = 0.80, 95% CI 0.52–1.14 higher vs. lowest tertile and OR = 0.94, 95% CI 0.87–1.02 linear trend). No clear association emerged for other food and beverage categories. No substantial difference was detected between partially and fully adjusted model. Graphical plots of the aforementioned associations, obtained using regression spline analysis, are depicted in Figure 1. These show a substantially linear trend in all selected categories, except for risk going toward the null at a very high intake (>60 g/day) of olive oil.

In sex and age stratified analyses (Table 4 and Supplementary Table S1), we observed a direct association between melanoma risk and consumption of ‘Cabbages’, ‘Chocolate, candy bars, etc.,’ and ‘Ice-cream’ in older subjects (≥50 years), particularly in men. On the other hand, the protective effect on melanoma risk yielded by ‘Legumes’ was higher in women (OR = 0.64, 95% CI 0.38–1.08 higher vs. lowest tertile and OR = 0.84, 95% CI 0.72–0.98 linear trend) and seemed to be limited to older subjects (OR = 0.75, 95% CI 0.47–1.21 higher vs. lowest tertile and OR = 0.87, 95% CI 0.77–0.98 linear trend). In addition, a sex and age specific direct association has emerged for ‘Dried fruits, nuts and seeds’ in men (OR = 1.34, 95% CI 0.78–2.31 highest vs. lowest and OR = 1.03, 95% CI 0.97–1.09 linear trend), especially in younger subjects (OR = 1.65, 95% CI 0.92–2.98 highest vs. lowest tertile and OR = 1.06, 95% CI 0.96–1.17 linear trend), and for ‘Fruit juices’ in women (OR = 1.91, 95% CI 1.16–3.15 highest vs. lowest tertile and OR = 1.02, 95% CI 1.00–1.04 linear trend).

Table 5 and Supplementary Table S2 summarize results for the analysis stratified by level of adherence to the Mediterranean diet (GMI score 0–4 vs. 5–9). In subjects with higher GMI score, no food item seemed to have a clear direct association with melanoma risk, except for ‘Processed meat’ and a small and statistically unstable relation for some types of ‘Sweets’ and ‘White wine’. In subjects with lower GMI score, the intake of ‘Meat and meat products’, especially ‘Red meat’, ‘Cheese’ and ‘Mushrooms’ appeared to qualify as a risk factor for melanoma. On the other hand, ‘Tomato’ consumption showed a protective effect.

## 4. Discussion

The main findings of our large population-based case-control study lay in an association between higher intake of cereal products, sweets, and cabbages and slightly increased melanoma risk. While higher consumption of legumes, olive oil, eggs, and onion and garlic was linked to decreased risk.

An association between increased cancer risk and high intake of foods such as cereal products and sweets, characterized by high contents of refined flours and sugars and therefore by a high glycemic index, has been observed at several sites such as colon-rectum, breast and endometrium [18,31,32], possibly due to increased postprandial glucose and insulin levels [33]. In addition, Gogas and colleagues suggest a possible role in melanoma development of high circulating levels of leptin, a factor involved in glucose metabolism and directly related with obesity, insulin levels and female sex [34]. Accordingly, we highlighted that a high intake of cereals and sweets could increase melanoma risk in our study population, especially in women, which confirms previous findings in the study population based on an assessment of the glycemic load of the diet [6].

High vegetable consumption has an established beneficial influence upon cancer risk, possibly including melanoma [35]. In our population, we found some indication of a protective effect on melanoma risk due to onion and garlic consumption as well as vegetables, particularly in women and in younger participants. On the other hand, cabbage consumption was associated with higher melanoma risk. This apparently conflicts with previous findings that highlighted a possible protective role of the intake of *Brassicas* species including all types of cabbages, broccoli, cauliflower and Brussels sprouts, through their high contents of glucosinolate compounds, especially isothiocyanates [36,37]. However, cabbages are also a possible source of heavy metals including cadmium [38], which has been associated to increased melanoma risk [39,40].

We found an inverse correlation between melanoma risk and consumption of legumes, olive oil and eggs. Eggs, with an average content of 3 µg/100 g, are among the major dietary sources of vitamin D, which could have a protective effect on melanoma risk [11]. In our population, however, the consumption of other foods rich in vitamin D such as fish, and dairy products, showed no clear relation with disease risk. Similarly, legumes, mainly beans and peas in our population, provide large quantities of folate, phytochemicals, sterols and several substances with putative antioxidant and anticancer properties such as glutathione, tocopherols and phenolic compounds, which might inhibit the process of melanoma carcinogenesis in both initiation and progression phases [41]. Olive oil consumption might have a protective role against cancer and other inflammation-related diseases [42]. The preventive activity of olive oil has been attributed to phenolic compounds. Several studies have demonstrated their ability to protect against DNA damage initiated by free radicals, inhibit proliferation and induce apoptosis in different tumor cell lines [43,44]. Another possible mechanism may be mediated by regulation of the ketogenic pathway, since it has been noted that the melanoma cells may promote proliferation and tumor growth by upregulating the expression of ketogenic enzymes [45].

In our study, we found an inverse correlation with melanoma risk in all age and sex groups, mostly in subjects with low GMI. When we stratified the analysis for level of adherence to the Mediterranean diet, we observed that in subjects with a high GMI score, no specific food consumption appeared to have a positive association with melanoma risk, except for processed meat and possibly some types of sweets and white wines. The traditional Mediterranean diet is associated with long life, lower prevalence of cardiovascular disease and cancers [46,47]. Its main components include a high intake of vegetables, fruit, olive oil, a moderate intake of fish and wine, and a low dairy and meat intake. Recently, it has been suggested that the low phosphorus content of the Mediterranean diet is linked to reduced cancer risk [48]. In addition, it may be that high adherence to the Mediterranean diet counteracts the unfavorable effects of other food categories. Conversely, in subjects with lower GMI score, the positive association between melanoma risk by meat and meat products (especially red meat), and mushrooms, appeared to be enhanced.

Some investigations suggested a protective effect on melanoma risk linked to fish and fruit intake [35], while citrus fruit consumption has been associated with an increased risk of melanoma [49]. In our study, none of these food categories were associated with melanoma risk. Similarly, we have not been able to detect a clear relation of meat and dairy products with disease risk, despite the positive association found in other studies with melanoma and cancer risk [35].

None of the beverages assessed in our study showed any clear effect on melanoma risk. This is partially at odds with previous studies, which found some negative and positive associations with coffee, fruit juices, and alcohol [15,16,50].

Our study was one of the largest population-based case-control studies investigating the association between diet and melanoma in Italy. A major strength of this investigation appears to be the validity and completeness of the EPIC food questionnaire. This had been specifically developed and validated in the Northern Italy population, including traditional seasoning and local dishes [21]. It also allowed us to conduct a very accurate assessment of exposure, including several pictures for portion anchors and the implementation of the Willet regression-residual method, and the exclusion of subjects with implausible total energy intake [51].

This study had some limitations, which must be acknowledged. We lacked information about type and number of dysplastic nevi, family history of melanoma and details about occupational history. However, in the most adjusted model, we took account of many other potential confounders such as phototype, sunburn history, education and BMI.

Moreover, our study was based on a case-control design. Therefore, we recognized that the study could have the general limitations concerning observational studies, i.e. unmeasured residual confounding, and in addition those inherent in the case-control design, such as the inability to assess disease incidence, the possibility of selection bias, and in cases recall bias. However, diet is not generally regarded by the general population as a risk factor for melanoma, thus making recall bias rather unlikely.

In addition, the questionnaire did not ascertain consumption of organic foods, such as organically-grown legumes, oil, and eggs. Therefore, we could not examine the association between organic food consumption and melanoma risk. Organic foods contain fewer pesticides and heavy metals [52], and organic food consumption has been associated with reduced cancer risk [53]. Therefore, our inability to account for consumption of such foods may have led to confounding. The impact of such bias is likely to be small because consumption of organic foods was uncommon during the study period [54].

Finally, the risk estimates we computed were generally statistically unstable, and such limited precision suggests additional caution in evaluating our findings, as well as the need to address these issues in larger studies.

## 5. Conclusions

In conclusion, our findings appear to confirm that dietary factors may play a role in melanoma risk, specifically suggesting potentially adverse effects of high consumption of cereal products, sweets and cabbages, and a possible protective role for legumes, olive oil, eggs, onion and garlic. In light of the rising melanoma incidence worldwide, and the current trend of some Western populations including the Italian one to deviate from the Mediterranean diet [55], these associations warrant further investigation for their potential public health and preventive medicine implications.

## Figures and Tables

**Table 1:** Baseline characteristics of study participants.

	Cases		Controls	
	*n*	(%)	*n*	(%)
Participants	380	(34.6)	719	(65.4)
Sex				
Male	175	(46.1)	319	(44.4)
Female	205	(53.9)	400	(55.6)
Age (years)				
<50	146	(38.4)	272	(37.8)
≥50	234	(61.6)	447	(62.2)
Education (years)				
≤5	91	(24.1)	170	(23.8)
6–8	95	(25.1)	176	(24.6)
9–13	136	(36.0)	266	(37.2)
≥14	56	(14.8)	103	(14.4)
Marital status				
Married	257	(67.6)	493	(68.7)
Unmarried/single	68	(17.9)	103	(14.3)
Divorced	23	(6.0)	48	(6.6)
Widowed	31	(8.2)	74	(10.3)
Unknown	1	(0.3)	1	(0.1)
Body mass index (kg/m^2^)				
≤19	31	(8.2)	45	(6.3)
20–24	164	(43.3)	306	(42.5)
25–29	133	(35.0)	287	(39.9)
≥30	52	(13.7)	81	(11.3)
Phototype ^a^				
I	105	(27.6)	109	(15.2)
II	136	(35.8)	238	(33.1)
III	122	(32.1)	312	(43.4)
IV	17	(4.5)	60	(8.3)
Sunburn history				
Never	182	(47.9)	452	(62.9)
Before 18 years	108	(28.4)	164	(22.8)
After 18 years	90	(23.7)	103	(14.3)
Greek Mediterranean index				
≤2	61	(16.1)	102	(14.2)
3–4	140	(36.8)	250	(34.8)
5–6	128	(33.7)	282	(39.2)
≥7	51	(13.4)	85	(11.8)
Glycemic load				
50th (IQR)	118.1	(89.7–150.1)	112.7	(87.0–148.1)
Vitamin C (mg/day)				
50th (IQR)	113.7	(78.0–154.1)	121.0	(83.66–162.6)
Vitamin D (mg/day)				
50th (IQR)	2.3	(1.7–3.1)	2.4	(1.8–3.4)
Energy (kcal/day)				
50th (IQR)	1.928	(1490–2443)	1.906	(1538–2365)

^a^ Phototype I, eyes/hair/skin light, high tendency to burn and never/moderate tan; Phototype II, eyes/hair/skin light, moderate tendency to burn and gradual tan or eyes/hair/skin brown, high tendency to burn and moderate tan; Phototype III, eyes/hair/skin brown, moderate/no tendency to burn and gradual/golden tan; Phototype IV, no tendency to burn and intense tan.

**Table 2:** Daily intake (g/day) of different food items or categories for cases and controls. Median (50th) and interquartile range (IQR) values calculated only in consumers.

Food Items	Cases			Controls		
	% Non Consumers	50th	IQR	% Non Consumers	50th	IQR
**Cereals and cereal products**	**0.0**	**162.6**	**119.2–201.9**	**0.0**	**147.8**	**110.5–190.4**
Pasta, other grain	0.5	56.0	37.8–80.0	1.3	55.9	37.1–76.9
Rice	17.9	3.9	2.4–6.9	20.4	3.8	1.9–6.3
Bread	5.5	86.4	45.6–124.0	8.1	76.4	44.4–113.7
Crackers, crispbread, salty snacks	9.5	4.6	2.2–17.4	10.4	4.5	2.1–16.3
**Meat and meat products**	**0.0**	**117.3**	**88.7–157.6**	**0.1**	**126.2**	**90.6–161.5**
Red meat	1.1	61.3	37.8–87.2	1.8	64.5	40.4–89.9
White meat	4.2	22.2	13.1–42.1	4.7	24.5	13.4–42.4
Processed meat	0.5	27.5	18.1–39.7	0.3	26.4	16.9–39.9
Offal	59.5	0.7	0.3–1.9	56.2	0.7	0.3–2.5
**Milk and dairy products**	**0.0**	**176.9**	**100.0–298.6**	**0.0**	**209.9**	**111.3–306.9**
Milk	35.3	110.8	30.3–224.3	32.7	134.4	36.5–223.4
Yogurt	36.3	27.4	13.8–70.6	33.8	41.2	13.5–108.3
Cheeses (including fresh cheeses)	0.0	42.4	27.9–58.0	0.8	38.4	27.1–54.9
**Eggs**	**0.8**	**11.4**	**7.1–17.1**	**2.1**	**13.5**	**7.9–20.1**
**Fish and seafood**	**2.4**	**28.8**	**15.9–42.7**	**2.1**	**29.9**	**17.7–45.2**
Fish	2.6	22.7	12.8–34.9	2.5	23.4	13.8–35.9
Crustaceans and molluscs	22.6	3.9	1.8–9.7	21.8	4.6	1.8–11.4
**Vegetables**	**0.0**	**136.7**	**98.8–197.5**	**0.0**	**147.4**	**105.1–204.0**
Leafy vegetables	0.5	23.7	12.3–43.5	0.1	25.5	14.6–44.2
Other vegetables	0.8	19.4	10.8–29.8	0.8	20.5	11.0–34.2
Tomatoes	0.8	53.3	31.8–85.4	0.7	54.3	33.5–86.5
Root vegetables	11.8	8.2	3.9–18.1	12.0	8.8	3.8–20.9
Cabbages	21.1	2.5	0.9–7.2	26.0	2.2	0.7–6.3
Mushrooms	22.4	1.6	0.8–3.3	20.7	1.7	0.8–3.8
Onion and garlic	0.5	10.8	6.4–20.5	0.3	12.6	7.3–21.9
**Legumes**	**4.5**	**13.9**	**7.82–22.4**	**3.6**	**15.5**	**8.3–24.1**
**Potatoes**	**1.3**	**19.3**	**12.0–31.6**	**1.4**	**20.4**	**12.5–32.7**
**Fresh fruit**	**0.3**	**244.7**	**167.8–357.5**	**0.8**	**257.1**	**174.4–361.8**
Citrus fruits	4.2	53.7	30.0–81.2	3.8	59.7	31.7–85.9
All other fruits	0.3	191.2	126.8–286.3	0.8	199.5	130.5–279.5
**Dried fruit, nuts and seeds**	**10.0**	**0.5**	**0.3–1.63**	**11.0**	**0.5**	**0.3–1.8**
**Sweets**	**1.3**	**84.6**	**56.7–113.1**	**1.9**	**80.0**	**55.3–113.5**
Chocolate, candy bars, etc.	25.0	4.4	2.6–8.5	31.4	3.6	2.1–7.4
Sugar, honey, jam, confectionery	13.2	14.9	7.2–27.61	12.2	14.5	7.8–27.6
Ice-cream	11.3	11.7	6.4–20.4	12.5	10.8	5.7–19.6
Cakes, pies and pastries	16.6	30.5	17.9–54.4	20.2	30.4	16.5–56.2
Biscuits, dry cakes	22.6	12.2	3.7–27.9	28.8	10.1	3.2–24.5
**Oils and fats**	**0.0**	**25.2**	**19.2–32.4**	**0.1**	**26.3**	**20.6–33.2**
Vegetable fats and non-olive oils	26.8	0.7	0.4–1.4	19.9	0.8	0.4–1.8
Olive oil	2.6	20.3	13.2–28.0	1.3	21.2	15.3–28.5
Butter and other animal fats	8.7	2.3	1.3–4.2	5.3	2.4	1.3–3.9
Coffee	8.2	79.1	49.7–116.6	9.9	83.9	49.3–123.2
Tea	39.7	21.8	2.7–86.5	47.3	10.6	1.7–65.4
Red wine	34.2	42.3	22.3–135.7	32.7	40.6	18.5–134.1
White wine	38.2	20.5	11.0–60.6	37.8	19.6	9.6–71.9
Aperitif wines and beers	31.6	26.1	13.3–41.2	30.2	26.0	13.8–50.7
Spirits and liqueurs	56.3	1.4	0.7–2.3	57.7	1.3	0.7–2.2
Fruit juices	38.7	45.9	25.7–107.3	39.6	43.5	21.8–102.0
Soft drinks	57.4	30.7	15.3–55.6	55.8	30.4	15.2–67.4

**Table 3:** Overall odds ratios (OR) and 95% confidence intervals (CI) for developing cutaneous malignant melanoma according to tertiles of daily intake (residual of regression on energy), obtained from conditional multiple logistic regression models. Linear trend for 10-g increments of daily intake.

Food Items	Cases/Controls	Median	OR ^a^	(95% CI)	OR ^b^	(95% CI)
Cereals and cereal products						
1st tertile (ref.)	111/249	92.1	1.00	-	1.00	-
2nd tertile	105/235	147.9	0.90	(0.64–1.27)	0.84	(0.58–1.21)
3nd tertile	164/235	215.0	1.57	(1.14–2.17)	1.32	(0.89–1.96)
*Linear trend*			*1.03*	*(1.01–1.05)*	*1.02*	*(1.00–1.05)*
Pasta, other grain						
1st tertile (ref.)	129/259	30.5	1.00	-	1.00	-
2nd tertile	123/230	55.5	1.13	(0.81–1.56)	1.06	(0.76–1.48)
3nd tertile	128/230	90.2	1.33	(0.95–1.85)	1.15	(0.82–1.63)
*Linear trend*			*1.04*	*(1.00–1.08)*	*1.02*	*(0.98–1.06)*
Rice						
1st tertile (ref.)	133/273	1.2	1.00	-	1.00	-
2nd tertile	122/223	3.8	1.14	(0.83–1.56)	1.11	(0.80–1.54)
3nd tertile	125/223	9.7	1.20	(0.88–1.64)	1.20	(0.88–1.65)
*Linear trend*			*1.01*	*(0.84–1.21)*	*1.02*	*(0.85–1.22)*
Bread						
1st tertile (ref.)	131/273	35.0	1.00	-	1.00	-
2nd tertile	101/223	76.7	0.89	(0.63–1.26)	0.89	(0.62–1.27)
3nd tertile	148/223	133.2	1.33	(0.97–1.82)	1.21	(0.83–1.77)
*Linear trend*			*1.02*	*(1.00–1.04)*	*1.01*	*(0.99–1.04)*
Crackers, crispbread, salty snacks						
1st tertile (ref.)	137/296	1.5	1.00	-	1.00	-
2nd tertile	127/212	4.5	1.44	(1.04–2.01)	1.37	(0.98–1.92)
3nd tertile	116/211	22.7	1.20	(0.87–1.65)	1.11	(0.79–1.55)
*Linear trend*			*1.04*	*(0.95–1.14)*	*1.01*	*(0.92–1.12)*
Meat and meat products						
1st tertile (ref.)	151/247	76.7	1.00	-	1.00	-
2nd tertile	117/236	125.9	0.85	(0.61–1.19)	0.83	(0.58–1.19)
3nd tertile	112/236	177.6	0.80	(0.57–1.12)	0.77	(0.53–1.14)
*Linear trend*			*0.99*	*(0.97–1.02)*	*1.00*	*(0.97–1.03)*
Red meat						
1st tertile (ref.)	150/253	32.9	1.00	-	1.00	-
2nd tertile	116/233	64.5	0.91	(0.66–1.26)	0.90	(0.64–1.26)
3nd tertile	114/233	104.9	0.90	(0.65–1.24)	0.87	(0.60–1.25)
*Linear trend*			*1.00*	*(0.96–1.03)*	*1.00*	*(0.96–1.04)*
White meat						
1st tertile (ref.)	144/258	10.2	1.00	-	1.00	-
2nd tertile	112/231	23.8	0.88	(0.64–1.22)	0.91	(0.65–1.26)
3nd tertile	124/230	51.4	0.97	(0.70–1.33)	1.09	(0.78–1.52)
*Linear trend*			*0.99*	*(0.94–1.04)*	*1.01*	*(0.95–1.06)*
Processed meat						
1st tertile (ref.)	130/257	13.6	1.00	-	1.00	-
2nd tertile	131/231	26.7	1.19	(0.85–1.67)	1.11	(0.79–1.57)
3nd tertile	119/231	46.5	1.03	(0.74–1.44)	1.01	(0.70–1.44)
*Linear trend*			*1.00*	*(0.93–1.06)*	*0.99*	*(0.92–1.06)*
Offal						
1st tertile (ref.)	192/354	0.2	1.00	-	1.00	-
2nd tertile	106/183	0.7	1.07	(0.76–1.51)	1.10	(0.77–1.55)
3nd tertile	82/182	4.0	0.89	(0.64–1.25)	0.98	(0.70–1.38)
*Linear trend* ^c^			*1.00*	*(0.97–1.03)*	*1.01*	*(0.98–1.04)*
Milk and dairy products						
1st tertile (ref.)	170/259	79.9	1.00	-	1.00	-
2nd tertile	104/230	210.4	0.65	(0.47–0.90)	0.67	(0.48–0.92)
3nd tertile	106/230	365.8	0.70	(0.50–0.97)	0.72	(0.51–1.01)
*Linear trend*			*1.00*	*(0.99–1.00)*	*1.00*	*(0.99–1.00)*
Milk						
1st tertile (ref.)	188/313	23.8	1.00	-	1.00	-
2nd tertile	82/203	131.5	0.68	(0.48–0.96)	0.71	(0.50–1.01)
3nd tertile	110/203	256.8	0.93	(0.68–1.29)	0.96	(0.69–1.33)
*Linear trend*			*1.00*	*(0.99–1.01)*	*1.00*	*(0.99–1.01)*
Yogurt						
1st tertile (ref.)	174/318	9.0	1.00	-	1.00	-
2nd tertile	133/201	36.8	1.21	(0.88–1.66)	1.28	(0.93–1.77)
3nd tertile	73/200	128.1	0.69	(0.48–0.98)	0.76	(0.53–1.09)
*Linear trend*			*0.98*	*(0.97–1.00)*	*0.99*	*(0.97–1.00)*
Cheeses (including fresh cheeses)						
1st tertile (ref.)	125/259	20.9	1.00	-	1.00	-
2nd tertile	118/230	38.7	1.03	(0.74–1.45)	1.01	(0.71–1.45)
3nd tertile	137/230	65.0	1.11	(0.80–1.54)	1.17	(0.81–1.70)
*Linear trend*			*1.03*	*(0.98–1.07)*	*1.03*	*(0.98–1.09)*
Eggs						
1st tertile (ref.)	176/249	6.2	1.00	-	1.00	-
2nd tertile	120/235	13.5	0.71	(0.52–0.96)	0.73	(0.53–0.99)
3nd tertile	84/235	23.4	0.53	(0.38–0.74)	0.58	(0.41–0.82)
*Linear trend* ^c^			*0.98*	*(0.97–0.99)*	*0.98*	*(0.97–1.00)*
Fish and seafood						
1st tertile (ref.)	143/250	13.6	1.00	-	1.00	-
2nd tertile	123/235	30.0	0.88	(0.64–1.20)	1.01	(0.72–1.43)
3nd tertile	114/234	54.6	0.77	(0.56–1.07)	1.11	(0.73–1.70)
*Linear trend*			*0.95*	*(0.90–1.00)*	*1.01*	*(0.94–1.08)*
Fish						
1st tertile (ref.)	141/251	10.7	1.00	-	1.00	-
2nd tertile	124/234	23.4	0.90	(0.66–1.23)	1.08	(0.77–1.51)
3nd tertile	115/234	43.9	0.81	(0.58–1.11)	1.24	(0.80–1.92)
*Linear trend*			*0.94*	*(0.88–1.01)*	*1.04*	*(0.94–1.14)*
Crustaceans and molluscs						
1st tertile (ref.)	162/290	1.3	1.00	-	1.00	-
2nd tertile	117/215	4.6	1.02	(0.74–1.42)	1.08	(0.77–1.50)
3nd tertile	101/214	14.9	0.85	(0.62–1.18)	0.97	(0.69–1.37)
*Linear trend* ^c^			*0.99*	*(0.97–1.00)*	*0.99*	*(0.98–1.01)*
Vegetables						
1st tertile (ref.)	148/243	87.3	1.00	-	1.00	-
2nd tertile	111/238	147.0	0.74	(0.54–1.02)	0.80	(0.57–1.14)
3nd tertile	121/238	235.3	0.81	(0.58–1.12)	0.95	(0.63–1.43)
*Linear trend*			*0.99*	*(0.97–1.00)*	*1.00*	*(0.98–1.02)*
Leafy vegetables						
1st tertile (ref.)	144/248	10.6	1.00	-	1.00	-
2nd tertile	121/236	25.2	0.80	(0.58–1.10)	0.85	(0.60–1.21)
3nd tertile	115/235	54.5	0.80	(0.57–1.11)	0.94	(0.64–1.37)
*Linear trend*			*0.98*	*(0.93–1.04)*	*1.02*	*(0.96–1.09)*
Other vegetables						
1st tertile (ref.)	137/250	8.4	1.00	-	1.00	-
2nd tertile	141/235	20.5	1.12	(0.81–1.55)	1.24	(0.88–1.73)
3nd tertile	102/234	40.6	0.81	(0.58–1.13)	0.96	(0.66–1.39)
*Linear trend*			*0.95*	*(0.88–1.02)*	*0.98*	*(0.90–1.07)*
Tomatoes						
1st tertile (ref.)	131/255	25.1	1.00	-	1.00	-
2nd tertile	130/232	54.1	1.08	(0.78–1.48)	1.13	(0.81–1.57)
3nd tertile	119/232	103.1	0.96	(0.70–1.32)	1.02	(0.72–1.46)
*Linear trend*			*0.99*	*(0.97–1.02)*	*1.00*	*(0.97–1.03)*
Root vegetables						
1st tertile (ref.)	145/284	2.6	1.00	-	1.00	-
2nd tertile	135/218	8.6	1.23	(0.88–1.70)	1.31	(0.93–1.83)
3nd tertile	100/217	28.9	0.82	(0.58–1.16)	0.90	(0.62–1.32)
*Linear trend* ^c^			*1.00*	*(0.99–1.00)*	*1.00*	*(0.99–1.01)*
Cabbages						
1st tertile (ref.)	140/297	0.4	1.00	-	1.00	-
2nd tertile	120/211	2.3	1.22	(0.89–1.67)	1.38	(1.00–1.92)
3nd tertile	120/211	8.9	1.25	(0.91–1.73)	1.52	(1.08–2.14)
*Linear trend* ^c^			*1.01*	*(0.99–1.03)*	*1.02*	*(1.00–1.04)*
Mushrooms						
1st tertile (ref.)	151/291	0.6	1.00	-	1.00	-
2nd tertile	130/214	1.6	1.25	(0.90–1.73)	1.34	(0.96–1.86)
3nd tertile	99/214	4.7	0.90	(0.64–1.25)	1.09	(0.76–1.55)
*Linear trend* ^c^			*0.97*	*(0.93–1.01)*	*0.99*	*(0.95–1.03)*
Onion and garlic						
1st tertile (ref.)	176/270	5.7	1.00	-	1.00	-
2nd tertile	98/225	12.5	0.67	(0.48–0.94)	0.70	(0.50–0.98)
3nd tertile	106/224	27.9	0.71	(0.51–1.00)	0.80	(0.56–1.14)
*Linear trend*			*0.92*	*(0.86–0.99)*	*0.94*	*(0.87–1.02)*
Legumes						
1st tertile (ref.)	148/256	6.0	1.00	-	1.00	-
2nd tertile	130/232	15.4	0.97	(0.71–1.34)	1.01	(0.73–1.41)
3nd tertile	102/231	29.6	0.71	(0.50–1.00)	0.77	(0.52–1.13)
*Linear trend*			*0.90*	*(0.82–0.98)*	*0.91*	*(0.82–1.00)*
Potatoes						
1st tertile (ref.)	142/262	9.5	1.00	-	1.00	-
2nd tertile	134/229	20.1	1.11	(0.80–1.53)	1.14	(0.82–1.58)
3nd tertile	104/228	41.6	0.81	(0.58–1.12)	0.83	(0.60–1.16)
*Linear trend*			*0.98*	*(0.92–1.04)*	*0.99*	*(0.93–1.05)*
Fresh fruit						
1st tertile (ref.)	138/245	137.9	1.00	-	1.00	-
2nd tertile	125/237	255.0	0.88	(0.63–1.22)	0.98	(0.69–1.39)
3nd tertile	117/237	411.6	0.83	(0.60–1.16)	1.09	(0.72–1.66)
*Linear trend*			*0.99*	*(0.98–1.00)*	*1.00*	*(0.99–1.01)*
Citrus fruits						
1st tertile (ref.)	150/255	19.5	1.00	-	1.00	-
2nd tertile	122/232	58.7	0.91	(0.66–1.26)	0.97	(0.70–1.36)
3nd tertile	108/232	104.5	0.77	(0.55–1.07)	0.93	(0.64–1.37)
*Linear trend*			*0.99*	*(0.96–1.01)*	*1.01*	*(0.97–1.04)*
All other fruits						
1st tertile (ref.)	140/245	103.1	1.00	-	1.00	-
2nd tertile	112/237	198.4	0.81	(0.58–1.14)	0.90	(0.64–1.27)
3nd tertile	128/237	319.7	0.88	(0.63–1.23)	1.13	(0.76–1.69)
*Linear trend*			*0.99*	*(0.98–1.00)*	*1.00*	*(0.99–1.01)*
Dried fruit, nuts and seeds						
1st tertile (ref.)	145/277	0.2	1.00	-	1.00	-
2nd tertile	123/221	0.5	1.16	(0.83–1.62)	1.21	(0.86–1.70)
3nd tertile	112/221	3.2	1.00	(0.72–1.38)	1.08	(0.77–1.52)
*Linear trend* ^c^			*1.01*	*(0.97–1.04)*	*1.01*	*(0.98–1.05)*
Sweets						
1st tertile (ref.)	128/258	42.3	1.00	-	1.00	-
2nd tertile	124/231	81.2	1.20	(0.85–1.70)	1.19	(0.84–1.69)
3nd tertile	128/230	130.7	1.21	(0.86–1.69)	1.22	(0.84–1.76)
*Linear trend*			*1.01*	*(0.98–1.03)*	*1.01*	*(0.98–1.04)*
Chocolate, candy bars, etc.						
1st tertile (ref.)	133/321	1.5	1.00	-	1.00	-
2nd tertile	121/199	3.7	1.54	(1.08–2.17)	1.50	(1.05–2.13)
3nd tertile	126/199	10.5	1.55	(1.12–2.13)	1.51	(1.09–2.09)
*Linear trend*			*1.06*	*(0.89–1.26)*	*1.05*	*(0.88–1.26)*
Sugar, honey, jam, confectionery						
1st tertile (ref.)	155/277	5.8	1.00	-	1.00	-
2nd tertile	112/221	14.8	0.98	(0.70–1.36)	1.00	(0.71–1.41)
3nd tertile	113/221	34.0	0.88	(0.63–1.23)	0.82	(0.58–1.16)
*Linear trend*			*1.00*	*(0.94–1.07)*	*0.98*	*(0.92–1.05)*
Ice-cream						
1st tertile (ref.)	139/275	4.3	1.00	-	1.00	-
2nd tertile	123/222	11.0	1.23	(0.89–1.70)	1.24	(0.89–1.71)
3nd tertile	118/222	25.7	1.08	(0.77–1.51)	1.12	(0.79–1.58)
*Linear trend*			*1.03*	*(0.94–1.13)*	*1.04*	*(0.94–1.14)*
Cakes, pies and pastries						
1st tertile (ref.)	152/309	12.0	1.00	-	1.00	-
2nd tertile	116/205	30.3	1.18	(0.84–1.67)	1.23	(0.87–1.74)
3nd tertile	112/205	70.8	1.14	(0.83–1.57)	1.26	(0.89–1.78)
*Linear trend*			*1.00*	*(0.97–1.03)*	*1.01*	*(0.98–1.05)*
Biscuits, dry cakes						
1st tertile (ref.)	147/300	2.3	1.00	-	1.00	-
2nd tertile	112/210	10.1	1.09	(0.78–1.51)	1.10	(0.79–1.53)
3nd tertile	121/209	31.8	1.22	(0.89–1.68)	1.14	(0.82–1.58)
*Linear trend*			*1.04*	*(0.96–1.13)*	*1.02*	*(0.95–1.11)*
Oils and fats						
1st tertile (ref.)	142/245	17.7	1.00	-	1.00	-
2nd tertile	124/237	26.3	0.83	(0.60–1.14)	0.85	(0.61–1.20)
3nd tertile	114/237	36.8	0.81	(0.59–1.13)	0.89	(0.59–1.34)
*Linear trend*			*0.92*	*(0.82–1.04)*	*0.95*	*(0.81–1.13)*
Vegetable fats and non-olive oils						
1st tertile (ref.)	180/322	0.3	1.00	-	1.00	-
2nd tertile	119/199	0.8	1.09	(0.77–1.54)	1.10	(0.77–1.57)
3nd tertile	81/198	6.0	0.75	(0.52–1.06)	0.72	(0.50–1.04)
*Linear trend* ^c^			*1.00*	*(0.98–1.02)*	*1.00*	*(0.98–1.03)*
Olive oil						
1st tertile (ref.)	147/248	12.1	1.00	-	1.00	-
2nd tertile	121/236	21.3	0.81	(0.59–1.11)	0.81	(0.58–1.14)
3nd tertile	112/235	32.4	0.74	(0.53–1.03)	0.77	(0.51–1.16)
*Linear trend*			*0.92*	*(0.82–1.03)*	*0.95*	*(0.81–1.10)*
Butter and other animal fats						
1st tertile (ref.)	164/282	0.9	1.00	-	1.00	-
2nd tertile	99/219	2.3	0.87	(0.63–1.21)	0.87	(0.62–1.21)
3nd tertile	117/218	4.8	0.94	(0.69–1.29)	0.91	(0.64–1.28)
*Linear trend* ^c^			*1.00*	*(0.96–1.04)*	*1.00*	*(0.96–1.05)*
Coffee						
1st tertile (ref.)	138/264	36.4	1.00	-	1.00	-
2nd tertile	138/228	83.5	1.20	(0.88–1.63)	1.22	(0.89–1.67)
3nd tertile	104/227	149.0	0.90	(0.65–1.24)	0.89	(0.64–1.24)
*Linear trend*			*0.99*	*(0.98–1.01)*	*0.99*	*(0.98–1.01)*
Tea						
1st tertile (ref.)	143/321	1.1	1.00	-	1.00	-
2nd tertile	116/199	12.3	1.31	(0.95–1.80)	1.32	(0.95–1.83)
3nd tertile	121/199	148.8	1.32	(0.96–1.81)	1.32	(0.96–1.82)
*Linear trend*			*1.00*	*(0.98–1.01)*	*1.00*	*(0.98–1.01)*
Red wine						
1st tertile (ref.)	168/323	13.0	1.00	-	1.00	-
2nd tertile	103/198	39.5	1.09	(0.76–1.57)	1.18	(0.81–1.71)
3nd tertile	109/198	206.7	1.13	(0.79–1.61)	1.17	(0.80–1.69)
*Linear trend*			*1.00*	*(0.99–1.01)*	*1.00*	*(0.99–1.01)*
White wine						
1st tertile (ref.)	165/333	6.4	1.00	-	1.00	-
2nd tertile	123/193	19.7	1.44	(1.01–2.06)	1.38	(0.95–1.99)
3nd tertile	92/193	128.1	1.03	(0.73–1.45)	1.05	(0.73–1.49)
*Linear trend*			*0.99*	*(0.98–1.01)*	*0.99*	*(0.98–1.01)*
Aperitif wines and beers						
1st tertile (ref.)	187/347	10.3	1.00	-	1.00	-
2nd tertile	103/186	26.2	0.94	(0.66–1.36)	0.93	(0.64–1.34)
3nd tertile	90/186	72.0	0.83	(0.58–1.19)	0.81	(0.56–1.18)
*Linear trend*			*0.99*	*(0.97–1.00)*	*0.99*	*(0.97–1.00)*
Spirits and liqueurs						
1st tertile (ref.)	204/372	0.5	1.00	-	1.00	-
2nd tertile	91/174	1.4	0.93	(0.64–1.36)	0.93	(0.63–1.36)
3nd tertile	85/173	4.5	0.92	(0.63–1.35)	0.94	(0.63–1.39)
*Linear trend* c			*0.99*	*(0.98–1.01)*	*0.93*	*(0.79–1.09)*
Fruit juices						
1st tertile (ref.)	172/331	15.6	1.00	-	1.00	-
2nd tertile	101/194	42.9	1.04	(0.73–1.48)	1.11	(0.77–1.59)
3nd tertile	107/194	136.1	1.07	(0.77–1.49)	1.29	(0.90–1.86)
*Linear trend*			*0.99*	*(0.98–1.01)*	*0.99*	*(0.98–1.01)*
Soft drinks						
1st tertile (ref.)	194/361	10.7	1.00	-	1.00	-
2nd tertile	101/179	30.7	1.04	(0.73–1.49)	1.00	(0.70–1.44)
3nd tertile	85/179	91.7	0.82	(0.58–1.17)	0.75	(0.52–1.08)
*Linear trend*			*0.99*	*(0.98–1.01)*	*0.99*	*(0.97–1.00)*

^a^ Adjusted for phototype, sunburn history, education, body mass index and non-alcohol energy, ^b^ Further adjusted for vitamin C and vitamin D intake, Greek Mediterranean index and glycemic index, ^c^ 1-g increments of daily intake.

**Table 4:** Overall adjusted ^a^ odds ratios (OR) and 95% confidence intervals (CI) for developing cutaneous malignant melanoma associated to 10-g increments of daily intake (residual of regression on energy) according to sex or age.

Cases/Controls (*n*)	Men		Women		<50 years		≥50 years	
	175/319		205/400		146/272		234/447	
	OR	(95% CI)	OR	(95% CI)	OR	(95% CI)	OR	(95% CI)
**Cereals and cereal products**	**1.03**	**(0.99–1.07)**	**1.02**	**(0.98–1.06)**	**1.02**	**(0.98–1.07)**	**1.03**	**(0.99–1.06)**
Pasta, other grain	1.01	(0.96–1.07)	1.03	(0.97–1.10)	1.00	(0.93–1.07)	1.02	(0.97–1.08)
Rice	1.01	(0.78–1.31)	1.04	(0.79–1.38)	0.98	(0.71–1.35)	1.08	(0.86–1.35)
Bread	1.03	(0.98–1.08)	1.00	(0.96–1.04)	1.04	(0.99–1.09)	1.01	(0.97–1.05)
Crackers, crispbread, salty snacks	0.98	(0.84–1.15)	1.05	(0.92–1.20)	0.89	(0.73–1.09)	1.06	(0.94–1.19)
**Meat and meat products**	**1.01**	**(0.97–1.06)**	**0.99**	**(0.95–1.03)**	**1.00**	**(0.96–1.04)**	**1.00**	**(0.96–1.03)**
Red meat	1.02	(0.97–1.07)	0.98	(0.92–1.03)	1.00	(0.94–1.06)	0.99	(0.94–1.04)
White meat	1.02	(0.94–1.10)	1.01	(0.93–1.09)	1.03	(0.94–1.12)	1.00	(0.93–1.08)
Processed meat	1.01	(0.91–1.12)	0.98	(0.88–1.09)	0.93	(0.83–1.05)	1.02	(0.92–1.13)
Offal ^b^	0.95	(0.90–1.01)	1.04	(1.00–1.09)	1.06	(0.99–1.14)	0.99	(0.95–1.03)
**Milk and dairy products**	**1.00**	**(0.98–1.01)**	**1.00**	**(0.99–1.01)**	**1.00**	**(0.99–1.01)**	**0.99**	**(0.98–1.00)**
Milk	1.00	(0.98–1.01)	1.00	(0.99–1.01)	1.01	(0.99–1.02)	0.99	(0.98–1.01)
Yogurt	0.99	(0.97–1.02)	0.98	(0.96–1.00)	0.97	(0.94–1.00)	0.99	(0.97–1.01)
Cheeses (including fresh cheeses)	1.06	(0.98–1.16)	1.02	(0.95–1.10)	1.02	(0.94–1.11)	1.07	(0.99–1.16)
**Eggs ^b^**	**0.98**	**(0.96–1.00)**	**0.98**	**(0.96–1.00)**	**0.99**	**(0.97–1.02)**	**0.98**	**(0.96–0.99)**
**Fish and seafood**	**0.99**	**(0.88–1.10)**	**1.03**	**(0.93–1.15)**	**0.96**	**(0.85–1.09)**	**1.02**	**(0.92–1.12)**
Fish	0.97	(0.84–1.12)	1.10	(0.96–1.26)	0.93	(0.79–1.11)	1.08	(0.95–1.22)
Crustaceans and molluscs ^b^	1.00	(0.98–1.03)	0.99	(0.97–1.01)	1.00	(0.98–1.02)	0.99	(0.96–1.01)
**Vegetables**	**0.98**	**(0.95–1.02)**	**1.00**	**(0.97–1.03)**	**1.01**	**(0.97–1.04)**	**0.99**	**(0.97–1.02)**
Leafy vegetables	0.97	(0.87–1.07)	1.04	(0.96–1.13)	0.99	(0.89–1.10)	1.06	(0.97–1.15)
Other vegetables	1.01	(0.87–1.17)	0.98	(0.88–1.08)	0.99	(0.85–1.16)	0.97	(0.87–1.07)
Tomatoes	0.98	(0.94–1.02)	1.01	(0.97–1.06)	1.02	(0.97–1.07)	0.98	(0.94–1.03)
Root vegetables ^b^	1.00	(0.99–1.01)	1.00	(0.99–1.01)	1.00	(0.99–1.01)	1.00	(0.98–1.01)
Cabbages ^b^	1.02	(0.99–1.05)	1.02	(0.99–1.05)	1.02	(0.99–1.06)	1.03	(1.00–1.06)
Mushrooms ^b^	0.97	(0.91–1.03)	1.00	(0.95–1.05)	1.07	(0.99–1.16)	0.95	(0.89–1.01)
Onion and garlic	0.93	(0.83–1.05)	0.94	(0.83–1.05)	0.94	(0.82–1.08)	0.94	(0.85–1.04)
**Legumes**	**0.97**	**(0.84–1.11)**	**0.84**	**(0.72–0.98)**	**1.03**	**(0.86–1.24)**	**0.87**	**(0.77–0.98)**
**Potatoes**	**1.02**	**(0.94–1.11)**	**0.93**	**(0.84–1.03)**	**1.09**	**(0.98–1.20)**	**0.91**	**(0.83–1.00)**
**Fresh fruit**	**1.01**	**(0.99–1.03)**	**0.99**	**(0.98–1.01)**	**0.99**	**(0.97–1.02)**	**1.01**	**(0.99–1.02)**
Citrus fruits	1.03	(0.98–1.08)	1.00	(0.96–1.05)	1.03	(0.97–1.09)	1.00	(0.95–1.04)
All other fruits	1.01	(0.99–1.04)	0.99	(0.97–1.01)	0.99	(0.96–1.01)	1.01	(0.99–1.03)
Dried fruit, nuts and seeds ^b^	1.03	(0.97–1.09)	0.99	(0.93–1.06)	1.06	(0.96–1.17)	1.01	(0.97–1.05)
**Sweets**	**0.99**	**(0.95–1.04)**	**1.02**	**(0.99–1.06)**	**1.01**	**(0.97–1.05)**	**1.01**	**(0.97–1.05)**
Chocolate, candy bars, etc.	1.02	(0.76–1.36)	1.08	(0.86–1.37)	0.97	(0.73–1.27)	1.11	(0.86–1.43)
Sugar, honey, jam, confectionery	0.94	(0.83–1.06)	1.00	(0.93–1.08)	1.06	(0.93–1.21)	0.96	(0.88–1.04)
Ice-cream	1.07	(0.94–1.23)	1.01	(0.88–1.16)	0.92	(0.79–1.07)	1.15	(1.01–1.31)
Cakes, pies and pastries	0.99	(0.93–1.05)	1.02	(0.97–1.07)	1.01	(0.96–1.07)	1.00	(0.95–1.06)
Biscuits, dry cakes	0.98	(0.85–1.13)	1.08	(0.98–1.20)	1.00	(0.88–1.14)	1.05	(0.95–1.17)
**Oils and fats**	**0.90**	**(0.70–1.15)**	**0.95**	**(0.75–1.20)**	**1.01**	**(0.77–1.33)**	**0.91**	**(0.73–1.13)**
Vegetable fats and non-olive oils	1.00	(0.97–1.03)	1.01	(0.97–1.04)	1.02	(0.98–1.06)	1.00	(0.97–1.03)
Olive oil	0.93	(0.74–1.18)	0.93	(0.76–1.15)	0.93	(0.72–1.22)	0.94	(0.77–1.14)
Butter and other animal fats ^b^	0.99	(0.93–1.05)	1.01	(0.95–1.08)	1.00	(0.93–1.07)	1.00	(0.94–1.06)
Coffee	1.00	(0.97–1.03)	0.99	(0.97–1.01)	1.01	(0.98–1.03)	0.98	(0.96–1.01)
Tea	1.01	(0.99–1.03)	0.99	(0.98–1.01)	1.00	(0.97–1.02)	1.00	(0.98–1.01)
Red wine	1.00	(0.98–1.01)	1.00	(0.97–1.02)	1.00	(0.97–1.03)	1.00	(0.98–1.01)
White wine	1.00	(0.98–1.01)	0.99	(0.96–1.01)	1.00	(0.97–1.03)	0.99	(0.98–1.01)
Aperitif wines and beers	1.00	(0.97–1.02)	0.97	(0.94–1.01)	0.98	(0.96–1.01)	0.99	(0.96–1.01)
Spirits and liqueurs ^b^	0.99	(0.98–1.01)	0.93	(0.86–1.00)	0.94	(0.90–1.00)	1.00	(0.98–1.02)
Fruit juices	0.97	(0.94–1.00)	1.02	(1.00–1.04)	1.00	(0.97–1.02)	1.01	(0.99–1.04)
Soft drinks	0.98	(0.96–1.01)	0.99	(0.97–1.01)	0.98	(0.96–1.00)	1.00	(0.97–1.02)

^a^ Adjusted for phototype, sunburn history, education, body mass index, non-alcohol energy, vitamin C, and vitamin D intake, Greek Mediterranean index and glycemic index, ^b^ 1-g increments of daily intake.

**Table 5:** Overall adjusted ^a^ odds ratios (OR) and 95% confidence intervals (CI) for developing cutaneous malignant melanoma associated to 10-g increments of daily intake (residual of regression on energy) according to adherence to the Mediterranean diet.

Cases/Controls (*n*)	Greek Mediterranean Index = 0–4		Greek Mediterranean Index = 5–9	
	201/352		179/367	
	OR	(95% CI)	OR	(95% CI)
**Cereals and cereal products**	**1.04**	**(0.99–1.08)**	**1.00**	**(0.95–1.06)**
Pasta, other grain	1.04	(0.97–1.13)	0.98	(0.91–1.06)
Rice	1.55	(1.04–2.31)	0.79	(0.54–1.15)
Bread	1.01	(0.96–1.06)	1.01	(0.95–1.07)
Crackers, crispbread, salty snacks	1.05	(0.87–1.27)	1.08	(0.90–1.28)
**Meat and meat products**	**1.07**	**(1.01–1.13)**	**1.00**	**(0.95–1.05)**
Red meat	1.09	(1.01–1.17)	0.95	(0.88–1.02)
White meat	1.10	(0.99–1.21)	0.99	(0.89–1.10)
Processed meat	0.91	(0.80–1.03)	1.20	(1.04–1.37)
Offal ^b^	1.02	(0.97–1.06)	0.97	(0.90–1.05)
**Milk and dairy products**	**1.00**	**(0.99–1.01)**	**0.98**	**(0.96–1.00)**
Milk	1.00	(0.99–1.02)	0.99	(0.96–1.01)
Yogurt	1.00	(0.97–1.03)	0.97	(0.93–1.01)
Cheeses (including fresh cheeses)	1.04	(0.96–1.14)	1.00	(0.89–1.12)
**Eggs ^b^**	**0.98**	**(0.95–1.01)**	**0.99**	**(0.96–1.01)**
**Fish and seafood**	**0.91**	**(0.76–1.09)**	**1.05**	**(0.92–1.19)**
Fish	0.92	(0.74–1.13)	1.08	(0.92–1.27)
Crustaceans and molluscs ^b^	0.99	(0.95–1.02)	1.00	(0.97–1.02)
**Vegetables**	**0.97**	**(0.93–1.01)**	**0.99**	**(0.95–1.03)**
Leafy vegetables	0.97	(0.85–1.10)	1.06	(0.95–1.18)
Other vegetables	1.06	(0.86–1.31)	0.85	(0.73–1.00)
Tomatoes	0.93	(0.87–0.99)	0.99	(0.94–1.05)
Root vegetables ^b^	1.00	(0.98–1.02)	1.00	(0.99–1.01)
Cabbages ^b^	1.03	(0.98–1.09)	1.03	(0.99–1.06)
Mushrooms ^b^	1.14	(1.03–1.25)	0.96	(0.89–1.03)
Onion and garlic	0.98	(0.81–1.19)	0.93	(0.82–1.04)
**Legumes**	**0.99**	**(0.82–1.20)**	**0.84**	**(0.70–1.01)**
**Potatoes**	**0.89**	**(0.78–1.02)**	**1.03**	**(0.92–1.14)**
**Fresh fruit**	**1.01**	**(0.98–1.03)**	**1.01**	**(0.99–1.03)**
Citrus fruits	1.06	(0.99–1.14)	1.03	(0.97–1.09)
All other fruits	1.00	(0.97–1.03)	1.00	(0.98–1.03)
**Dried fruit, nuts and seeds ^b^**	**1.03**	**(0.94–1.14)**	**1.00**	**(0.95–1.04)**
**Sweets**	**0.99**	**(0.95–1.04)**	**1.00**	**(0.96–1.05)**
Chocolate, candy bars, etc.	0.90	(0.67–1.23)	1.24	(0.89–1.72)
Sugar, honey, jam, confectionery	1.13	(0.97–1.31)	0.95	(0.87–1.05)
Ice-cream	0.96	(0.81–1.14)	1.07	(0.88–1.31)
Cakes, pies and pastries	0.98	(0.92–1.05)	1.02	(0.96–1.09)
Biscuits, dry cakes	0.96	(0.84–1.10)	1.00	(0.86–1.17)
**Oils and fats**	**0.88**	**(0.63–1.25)**	**0.91**	**(0.68–1.22)**
Vegetable fats and non-olive oils ^b^	1.01	(0.97–1.06)	1.00	(0.96–1.04)
Olive oil	0.77	(0.56–1.07)	0.97	(0.75–1.25)
Butter and other animal fats ^b^	1.06	(0.98–1.15)	0.97	(0.88–1.07)
Coffee	1.01	(0.98–1.03)	0.99	(0.96–1.02)
Tea	0.99	(0.96–1.01)	0.99	(0.97–1.01)
Red wine	0.98	(0.96–1.01)	0.99	(0.97–1.02)
White wine	0.99	(0.96–1.02)	1.03	(1.00–1.07)
Aperitif wines and beers	0.98	(0.96–1.01)	1.01	(0.98–1.05)
Spirits and liqueurs ^b^	0.99	(0.96–1.02)	1.00	(0.97–1.02)
Fruit juices	1.01	(0.98–1.05)	0.99	(0.96–1.02)
Soft drinks	1.00	(0.98–1.01)	0.99	(0.94–1.03)

^a^ Adjusted for phototype, sunburn history, education, body mass index, no-alcohol energy, vitamin C and vitamin D intake, Greek Mediterranean index and glycemic index, ^b^ 1-g increments of daily intake.

## Supplementary Materials

The following are available online at https://www.mdpi.com/2072-6643/11/9/2206/s1, Table S1. Overall adjusted odds ratios (OR) and 95% confidence intervals (CI) for developing cutaneous malignant melanoma according to tertiles of daily intake by sex or age. Lowest tertile as referent. Sex- and age-specific tertiles of food intake obtained from residual of regression on energy. Table S2. Overall adjusted ^a^ odds ratios (OR) and 95% confidence intervals (CI) for developing cutaneous malignant melanoma according to tertiles of daily intake by level of adherence to the Mediterranean diet. Lowest tertile as referent. Greek Mediterranean Index specific tertiles of food intake obtained from residual of regression on energy.
