# Supplementary material for: Food and Beverage Consumption and Melanoma Risk: A Population-Based Case-Control Study in Northern Italy
Source: Nutrients. 2019 Sep 12;11(9):2206. doi: 10.3390/nu11092206 (PMC6769978; doi:10.3390/nu11092206)
Supplement: Supplementary file 1 [file nutrients-11-02206-s001.pdf]

Supplemental Table S1. Overall adjusted[a] odds ratios (OR) and 95% confidence intervals (CI) for developing CMM according to tertiles of daily intake by sex or age. Lowest tertile as referent. Sex- and age-specific tertiles of food intake obtained from residual of regression on energy.

	Men		Women		<50 years		≥50 years	
Cases/controls (N)	175/319		205/400		146/272		234/447	
	OR	(95% CI)	OR	(95% CI)	OR	(95% CI)	OR	(95% CI)
Cereals and cereal products								
2nd tertile	0.89	(0.50 - 1.58)	1.00	(0.62 - 1.62)	0.96	(0.52 - 1.75)	0.95	(0.60 - 1.49)
3nd tertile	1.30	(0.71 - 2.39)	1.44	(0.85 - 2.44)	1.31	(0.70 - 2.42)	1.40	(0.85 - 2.32)
Pasta, other grain								
2nd tertile	1.03	(0.62 - 1.71)	1.11	(0.72 - 1.72)	1.11	(0.66 - 1.87)	0.97	(0.63 - 1.50)
3nd tertile	1.05	(0.62 - 1.79)	1.13	(0.71 - 1.79)	1.03	(0.59 - 1.82)	1.05	(0.66 - 1.67)
Rice								
2nd tertile	0.80	(0.48 - 1.33)	1.57	(1.00 - 2.45)	0.98	(0.59 - 1.62)	1.24	(0.81 - 1.89)
3nd tertile	0.99	(0.61 - 1.62)	1.54	(0.99 - 2.42)	1.16	(0.69 - 1.96)	1.39	(0.92 - 2.09)
Bread								
2nd tertile	0.90	(0.52 - 1.56)	0.82	(0.50 - 1.36)	0.82	(0.45 - 1.51)	0.86	(0.54 - 1.37)
3nd tertile	1.32	(0.72 - 2.41)	1.28	(0.77 - 2.12)	1.17	(0.64 - 2.14)	1.34	(0.82 - 2.17)
Crackers, crispbread, salty snacks								
2nd tertile	1.74	(1.03 - 2.92)	0.96	(0.60 - 1.54)	1.68	(0.96 - 2.92)	1.16	(0.75 - 1.79)
3nd tertile	1.37	(0.79 - 2.38)	0.96	(0.62 - 1.47)	0.95	(0.54 - 1.69)	1.19	(0.78 - 1.81)
Meat and meat products								
2nd tertile	0.79	(0.45 - 1.39)	0.74	(0.44 - 1.22)	0.86	(0.47 - 1.54)	0.73	(0.45 - 1.16)
3nd tertile	0.76	(0.42 - 1.37)	0.89	(0.53 - 1.48)	0.90	(0.49 - 1.66)	0.68	(0.42 - 1.12)
Red meat								
2nd tertile	0.88	(0.53 - 1.47)	0.87	(0.56 - 1.37)	0.96	(0.55 - 1.66)	0.70	(0.45 - 1.11)
3nd tertile	0.92	(0.52 - 1.63)	0.90	(0.56 - 1.46)	1.09	(0.63 - 1.91)	0.75	(0.46 - 1.21)
White meat								
2nd tertile	0.67	(0.39 - 1.14)	1.23	(0.79 - 1.93)	0.87	(0.49 - 1.52)	1.04	(0.68 - 1.60)
3nd tertile	0.99	(0.59 - 1.66)	1.30	(0.81 - 2.11)	1.24	(0.70 - 2.20)	1.03	(0.66 - 1.58)
Processed meat								
2nd tertile	1.15	(0.67 - 2.00)	1.13	(0.71 - 1.80)	1.21	(0.67 - 2.19)	1.25	(0.80 - 1.95)
3nd tertile	1.32	(0.74 - 2.35)	0.92	(0.57 - 1.49)	0.99	(0.54 - 1.81)	1.04	(0.66 - 1.63)
Offal								
2nd tertile	1.19	(0.70 - 2.04)	0.97	(0.59 - 1.59)	0.93	(0.48 - 1.77)	1.29	(0.85 - 1.97)
3nd tertile	0.72	(0.40 - 1.29)	1.35	(0.86 - 2.10)	1.47	(0.83 - 2.62)	0.77	(0.49 - 1.21)
Milk and dairy products								
2nd tertile	1.21	(0.74 - 1.99)	0.78	(0.50 - 1.23)	0.66	(0.37 - 1.18)	0.66	(0.44 - 1.01)
3nd tertile	0.95	(0.56 - 1.62)	0.88	(0.55 - 1.40)	1.30	(0.76 - 2.23)	0.56	(0.36 - 0.88)
Milk								
2nd tertile	0.69	(0.41 - 1.19)	0.65	(0.41 - 1.04)	1.09	(0.61 - 1.96)	0.60	(0.39 - 0.93)
3nd tertile	0.87	(0.52 - 1.46)	1.05	(0.68 - 1.62)	1.43	(0.83 - 2.46)	0.73	(0.47 - 1.13)
Yogurt								
2nd tertile	1.19	(0.70 - 2.01)	0.86	(0.57 - 1.30)	0.88	(0.53 - 1.49)	1.56	(1.02 - 2.36)
3nd tertile	1.02	(0.61 - 1.71)	0.68	(0.42 - 1.09)	0.60	(0.33 - 1.09)	0.87	(0.55 - 1.38)
Cheeses (including fresh cheeses)								
2nd tertile	0.87	(0.49 - 1.53)	0.98	(0.61 - 1.60)	1.24	(0.67 - 2.27)	0.88	(0.56 - 1.39)
3nd tertile	1.77	(0.98 - 3.21)	0.88	(0.52 - 1.46)	1.49	(0.83 - 2.65)	1.19	(0.75 - 1.91)
Eggs								
2nd tertile	0.89	(0.55 - 1.45)	0.82	(0.54 - 1.26)	1.10	(0.66 - 1.82)	0.67	(0.44 - 1.02)
3nd tertile	0.66	(0.39 - 1.12)	0.54	(0.33 - 0.88)	0.81	(0.46 - 1.43)	0.56	(0.36 - 0.88)
Fish and seafood								
2nd tertile	0.93	(0.55 - 1.57)	1.23	(0.77 - 1.97)	0.73	(0.41 - 1.29)	1.20	(0.78 - 1.85)
3nd tertile	0.98	(0.51 - 1.90)	1.41	(0.77 - 2.57)	1.04	(0.54 - 2.02)	1.20	(0.71 - 2.03)
Fish								
2nd tertile	0.98	(0.59 - 1.63)	1.19	(0.75 - 1.89)	0.75	(0.43 - 1.30)	1.15	(0.75 - 1.74)
3nd tertile	1.12	(0.57 - 2.19)	1.61	(0.89 - 2.93)	0.97	(0.49 - 1.94)	1.23	(0.72 - 2.10)
Crustaceans and molluscs								

2nd tertile	1.40	(0.83 - 2.35)	1.07	(0.69 - 1.68)	0.87	(0.51 - 1.49)	1.09	(0.70 - 1.68)
3rd tertile	1.03	(0.60 - 1.78)	1.01	(0.63 - 1.63)	0.75	(0.43 - 1.29)	0.96	(0.62 - 1.49)
Vegetables								
2nd tertile	0.68	(0.40 - 1.17)	0.95	(0.60 - 1.50)	0.68	(0.39 - 1.17)	0.87	(0.56 - 1.35)
3rd tertile	0.72	(0.39 - 1.33)	0.85	(0.49 - 1.48)	0.87	(0.48 - 1.59)	1.01	(0.62 - 1.64)
Leafy vegetables								
2nd tertile	0.75	(0.44 - 1.27)	1.15	(0.73 - 1.83)	1.05	(0.63 - 1.75)	0.77	(0.49 - 1.20)
3rd tertile	0.64	(0.35 - 1.15)	1.25	(0.73 - 2.13)	0.90	(0.48 - 1.68)	1.05	(0.66 - 1.67)
Other vegetables								
2nd tertile	0.95	(0.57 - 1.59)	1.15	(0.72 - 1.83)	1.21	(0.69 - 2.11)	1.28	(0.84 - 1.97)
3rd tertile	0.97	(0.55 - 1.70)	0.84	(0.51 - 1.39)	1.18	(0.65 - 2.14)	0.96	(0.61 - 1.52)
Tomatoes								
2nd tertile	1.00	(0.60 - 1.66)	1.06	(0.67 - 1.67)	1.22	(0.70 - 2.13)	1.05	(0.69 - 1.59)
3rd tertile	0.97	(0.57 - 1.65)	1.01	(0.62 - 1.63)	1.29	(0.73 - 2.28)	0.91	(0.59 - 1.42)
Root vegetables								
2nd tertile	1.13	(0.67 - 1.91)	1.07	(0.67 - 1.73)	1.59	(0.90 - 2.79)	0.99	(0.63 - 1.54)
3rd tertile	1.18	(0.67 - 2.09)	0.87	(0.53 - 1.43)	1.23	(0.67 - 2.28)	0.83	(0.52 - 1.33)
Cabbages								
2nd tertile	1.27	(0.75 - 2.14)	1.27	(0.80 - 1.99)	1.07	(0.62 - 1.87)	1.60	(1.03 - 2.48)
3rd tertile	2.09	(1.24 - 3.51)	1.21	(0.76 - 1.94)	1.52	(0.83 - 2.76)	1.80	(1.16 - 2.77)
Mushrooms								
2nd tertile	1.61	(0.93 - 2.78)	1.34	(0.86 - 2.08)	1.56	(0.87 - 2.79)	1.23	(0.81 - 1.85)
3rd tertile	1.18	(0.68 - 2.02)	1.12	(0.69 - 1.80)	1.67	(0.95 - 2.93)	0.87	(0.55 - 1.38)
Onion and garlic								
2nd tertile	0.63	(0.37 - 1.09)	0.84	(0.53 - 1.34)	1.15	(0.68 - 1.96)	0.56	(0.36 - 0.87)
3rd tertile	0.72	(0.41 - 1.24)	0.81	(0.50 - 1.33)	0.92	(0.51 - 1.66)	0.78	(0.50 - 1.21)
Legumes								
2nd tertile	1.36	(0.81 - 2.28)	0.85	(0.55 - 1.30)	0.79	(0.47 - 1.35)	1.08	(0.71 - 1.64)
3rd tertile	1.03	(0.55 - 1.93)	0.64	(0.38 - 1.08)	0.89	(0.51 - 1.57)	0.75	(0.47 - 1.21)
Potatoes								
2nd tertile	0.82	(0.49 - 1.38)	1.40	(0.89 - 2.19)	1.38	(0.78 - 2.42)	0.97	(0.64 - 1.47)
3rd tertile	1.01	(0.62 - 1.65)	0.70	(0.43 - 1.12)	1.31	(0.76 - 2.27)	0.60	(0.38 - 0.95)
Fruits								
2nd tertile	0.84	(0.49 - 1.44)	0.82	(0.51 - 1.32)	1.08	(0.64 - 1.84)	0.95	(0.60 - 1.51)
3rd tertile	1.23	(0.64 - 2.36)	0.77	(0.43 - 1.36)	1.04	(0.52 - 2.10)	1.05	(0.62 - 1.78)
Citrus fruits								
2nd tertile	1.27	(0.73 - 2.19)	1.00	(0.64 - 1.56)	1.35	(0.79 - 2.30)	0.85	(0.55 - 1.31)
3rd tertile	0.91	(0.48 - 1.71)	1.12	(0.68 - 1.84)	0.91	(0.47 - 1.75)	0.92	(0.57 - 1.50)
All other fruits								
2nd tertile	0.85	(0.50 - 1.46)	0.93	(0.59 - 1.46)	0.79	(0.45 - 1.40)	1.10	(0.70 - 1.70)
3rd tertile	1.40	(0.74 - 2.64)	0.79	(0.46 - 1.37)	0.93	(0.47 - 1.83)	1.29	(0.78 - 2.12)
Dried fruit, nuts and seeds								
2nd tertile	1.74	(1.03 - 2.93)	0.93	(0.59 - 1.46)	1.18	(0.66 - 2.13)	1.03	(0.68 - 1.57)
3rd tertile	1.34	(0.78 - 2.31)	0.95	(0.61 - 1.49)	1.65	(0.92 - 2.98)	0.94	(0.62 - 1.42)
Sweets								
2nd tertile	1.02	(0.59 - 1.74)	1.51	(0.95 - 2.40)	1.55	(0.85 - 2.85)	0.88	(0.56 - 1.38)
3rd tertile	1.06	(0.59 - 1.91)	1.29	(0.80 - 2.09)	1.50	(0.81 - 2.77)	0.98	(0.62 - 1.55)
Chocolate, candy bars, etc.								
2nd tertile	1.45	(0.85 - 2.49)	1.43	(0.89 - 2.29)	1.20	(0.65 - 2.19)	1.45	(0.92 - 2.29)
3rd tertile	1.60	(0.96 - 2.66)	1.72	(1.11 - 2.66)	1.76	(1.04 - 2.96)	1.50	(0.99 - 2.27)
Sugar, honey, jam, confectionery								
2nd tertile	1.07	(0.64 - 1.80)	0.95	(0.59 - 1.52)	1.45	(0.80 - 2.62)	0.84	(0.54 - 1.32)
3rd tertile	0.71	(0.42 - 1.20)	0.88	(0.54 - 1.42)	1.19	(0.68 - 2.08)	0.68	(0.43 - 1.08)
Ice-cream								
2nd tertile	1.17	(0.70 - 1.96)	0.99	(0.65 - 1.53)	1.38	(0.81 - 2.35)	1.15	(0.75 - 1.77)
3rd tertile	1.23	(0.72 - 2.09)	0.96	(0.61 - 1.52)	0.91	(0.51 - 1.62)	1.44	(0.93 - 2.24)
Cakes, pies and pastries								
2nd tertile	1.68	(0.94 - 3.00)	1.09	(0.68 - 1.74)	1.55	(0.88 - 2.75)	1.37	(0.87 - 2.16)
3rd tertile	1.21	(0.72 - 2.05)	1.25	(0.78 - 2.01)	1.48	(0.84 - 2.62)	1.09	(0.69 - 1.71)
Biscuits, dry cakes								
2nd tertile	0.94	(0.55 - 1.60)	1.37	(0.88 - 2.13)	1.28	(0.76 - 2.16)	1.15	(0.74 - 1.78)
3rd tertile	0.89	(0.52 - 1.52)	1.53	(0.99 - 2.35)	1.12	(0.65 - 1.92)	1.21	(0.79 - 1.84)
Oils and fats								
2nd tertile	0.95	(0.56 - 1.60)	0.67	(0.42 - 1.08)	0.91	(0.53 - 1.56)	0.96	(0.63 - 1.47)

3rd tertile	0.68	(0.35 - 1.33)	0.93	(0.54 - 1.61)	0.82	(0.44 - 1.53)	1.04	(0.64 - 1.67)
Vegetables fats and non-olive oils								
2nd tertile	1.00	(0.57 - 1.75)	1.30	(0.81 - 2.10)	1.02	(0.58 - 1.81)	1.06	(0.68 - 1.67)
3rd tertile	0.52	(0.28 - 0.95)	0.89	(0.55 - 1.43)	0.98	(0.55 - 1.76)	0.73	(0.46 - 1.15)
Olive oil								
2nd tertile	1.00	(0.59 - 1.72)	0.67	(0.42 - 1.06)	1.03	(0.61 - 1.73)	0.72	(0.47 - 1.11)
3rd tertile	0.63	(0.32 - 1.25)	0.89	(0.53 - 1.48)	0.83	(0.45 - 1.52)	0.85	(0.54 - 1.35)
Butter and other animal fats								
2nd tertile	0.65	(0.39 - 1.10)	1.25	(0.80 - 1.97)	0.88	(0.50 - 1.56)	0.89	(0.58 - 1.36)
3rd tertile	1.16	(0.71 - 1.90)	0.75	(0.46 - 1.23)	0.84	(0.48 - 1.47)	0.96	(0.61 - 1.49)
Coffee								
2nd tertile	1.31	(0.80 - 2.14)	0.94	(0.61 - 1.44)	1.42	(0.84 - 2.40)	1.01	(0.67 - 1.52)
3rd tertile	1.11	(0.66 - 1.84)	0.74	(0.47 - 1.16)	1.53	(0.89 - 2.66)	0.67	(0.44 - 1.02)
Tea								
2nd tertile	1.62	(0.95 - 2.75)	1.13	(0.74 - 1.75)	1.10	(0.65 - 1.89)	1.58	(1.03 - 2.42)
3rd tertile	1.59	(0.97 - 2.61)	1.22	(0.78 - 1.90)	1.44	(0.85 - 2.45)	1.30	(0.86 - 1.97)
Red wine								
2nd tertile	1.30	(0.78 - 2.16)	1.10	(0.65 - 1.87)	1.24	(0.64 - 2.39)	1.16	(0.75 - 1.79)
3rd tertile	0.97	(0.56 - 1.67)	1.00	(0.61 - 1.64)	1.30	(0.72 - 2.36)	0.97	(0.62 - 1.52)
White wine								
2nd tertile	1.03	(0.61 - 1.77)	0.98	(0.58 - 1.64)	1.02	(0.56 - 1.88)	1.29	(0.83 - 2.00)
3rd tertile	1.08	(0.64 - 1.81)	0.93	(0.57 - 1.53)	1.15	(0.64 - 2.07)	0.94	(0.61 - 1.46)
Aperitif wines and beers								
2nd tertile	1.67	(0.97 - 2.88)	0.61	(0.36 - 1.03)	1.09	(0.59 - 2.00)	0.98	(0.61 - 1.57)
3rd tertile	1.02	(0.57 - 1.83)	0.53	(0.31 - 0.91)	0.66	(0.36 - 1.22)	0.81	(0.50 - 1.31)
Spirits and liqueurs								
2nd tertile	0.81	(0.43 - 1.52)	0.99	(0.58 - 1.68)	1.10	(0.56 - 2.14)	0.76	(0.46 - 1.25)
3rd tertile	1.25	(0.72 - 2.18)	0.93	(0.53 - 1.63)	1.02	(0.54 - 1.94)	0.83	(0.49 - 1.41)
Fruit juices								
2nd tertile	1.30	(0.75 - 2.26)	1.14	(0.68 - 1.90)	0.97	(0.55 - 1.71)	1.05	(0.63 - 1.73)
3rd tertile	0.89	(0.49 - 1.62)	1.91	(1.16 - 3.15)	1.14	(0.58 - 2.24)	1.58	(1.00 - 2.50)
Soft drinks								
2nd tertile	0.86	(0.50 - 1.48)	1.10	(0.67 - 1.82)	0.88	(0.51 - 1.51)	1.05	(0.66 - 1.68)
3rd tertile	0.71	(0.39 - 1.28)	0.77	(0.46 - 1.27)	0.44	(0.23 - 0.83)	1.22	(0.75 - 1.97)

[a]Adjusted for phototype, sunburn history, education, body mass index, non-alcohol energy, vitamin C and vitamin D intake, Greek Mediterranean index and glycemic index.

Supplemental Table S2. Overall adjusted[a] odds ratios (OR) and 95% confidence intervals (CI) for developing CMM according to tertiles of daily intake by level of adherence to the Mediterranean diet. Lowest tertile as referent. Greek Mediterranean Index specific tertiles of food intake obtained from residual of regression on energy.

	**Greek Mediterranean Index = 0-4**		Greek Mediterranean Index = 5-9	
**Cases/controls (N)**	**201/352**		179/367	
	OR	(95% CI)	OR	(95% CI)
Cereals and cereal products				
2nd tertile	1.16	(0.60 - 2.24)	0.80	(0.40 - 1.60)
3rd tertile	1.78	(0.89 - 3.54)	1.10	(0.51 - 2.38)
Pasta, other grain				
2nd tertile	1.56	(0.83 - 2.94)	0.80	(0.42 - 1.53)
3rd tertile	1.05	(0.56 - 1.99)	1.00	(0.54 - 1.85)
Rice				
2nd tertile	1.13	(0.64 - 1.99)	0.98	(0.53 - 1.83)
3rd tertile	1.66	(0.93 - 2.95)	0.97	(0.54 - 1.73)
Bread				
2nd tertile	0.77	(0.41 - 1.45)	0.90	(0.47 - 1.74)
3rd tertile	1.34	(0.70 - 2.55)	1.22	(0.55 - 2.73)
Crackers, crispbread, salty snacks				
2nd tertile	2.40	(1.25 - 4.60)	1.23	(0.66 - 2.31)
3rd tertile	1.79	(0.94 - 3.41)	1.34	(0.72 - 2.50)
Meat and meat products				
2nd tertile	1.16	(0.60 - 2.26)	0.97	(0.51 - 1.84)
3rd tertile	1.73	(0.85 - 3.51)	0.54	(0.26 - 1.14)
Red meat				

2nd tertile	1.49	(0.81 - 2.73)	0.85	(0.45 - 1.61)
3nd tertile	1.82	(0.93 - 3.57)	0.52	(0.25 - 1.06)
White meat				
2nd tertile	1.71	(0.94 - 3.10)	0.91	(0.49 - 1.70)
3nd tertile	2.28	(1.19 - 4.38)	0.95	(0.52 - 1.76)
Processed meat				
2nd tertile	1.20	(0.63 - 2.27)	3.30	(1.62 - 6.72)
3nd tertile	0.74	(0.40 - 1.37)	3.03	(1.52 - 6.03)
Offal				
2nd tertile	0.98	(0.52 - 1.85)	1.10	(0.59 - 2.05)
3nd tertile	1.21	(0.67 - 2.19)	0.78	(0.39 - 1.56)
Milk and dairy products				
2nd tertile	0.57	(0.32 - 1.02)	0.64	(0.34 - 1.22)
3nd tertile	1.01	(0.57 - 1.80)	0.49	(0.25 - 0.97)
Milk				
2nd tertile	0.57	(0.31 - 1.05)	0.53	(0.28 - 1.02)
3nd tertile	1.07	(0.60 - 1.92)	0.80	(0.43 - 1.47)
Yogurt				
2nd tertile	1.11	(0.60 - 2.03)	1.20	(0.66 - 2.15)
3nd tertile	0.84	(0.44 - 1.61)	0.62	(0.32 - 1.21)
Cheeses (including fresh cheeses)				
2nd tertile	1.27	(0.67 - 2.40)	0.79	(0.41 - 1.54)
3nd tertile	1.60	(0.84 - 3.05)	1.60	(0.79 - 3.23)
Eggs				
2nd tertile	0.79	(0.46 - 1.34)	0.62	(0.32 - 1.17)
3nd tertile	0.51	(0.27 - 0.98)	0.64	(0.34 - 1.20)
Fish and seafood				
2nd tertile	0.92	(0.51 - 1.64)	1.83	(0.93 - 3.60)
3nd tertile	0.63	(0.30 - 1.32)	1.42	(0.66 - 3.04)
Fish				
2nd tertile	0.70	(0.38 - 1.29)	1.60	(0.83 - 3.09)
3nd tertile	0.78	(0.36 - 1.67)	1.70	(0.80 - 3.63)
Crustaceans and molluscs				
2nd tertile	1.48	(0.83 - 2.63)	0.83	(0.43 - 1.61)
3nd tertile	0.76	(0.42 - 1.39)	0.79	(0.42 - 1.49)
Vegetables				
2nd tertile	0.48	(0.25 - 0.91)	0.72	(0.38 - 1.38)
3nd tertile	0.47	(0.23 - 0.96)	0.80	(0.39 - 1.66)
Leafy vegetables				
2nd tertile	0.49	(0.25 - 0.95)	0.72	(0.35 - 1.48)
3nd tertile	0.84	(0.43 - 1.66)	1.33	(0.66 - 2.68)
Other vegetables				
2nd tertile	1.29	(0.71 - 2.35)	0.78	(0.42 - 1.44)
3nd tertile	1.42	(0.70 - 2.86)	0.51	(0.26 - 1.00)
Tomatoes				
2nd tertile	0.98	(0.54 - 1.78)	0.62	(0.34 - 1.13)
3nd tertile	0.53	(0.27 - 1.06)	0.80	(0.43 - 1.47)
Root vegetables				
2nd tertile	1.12	(0.60 - 2.08)	1.22	(0.64 - 2.32)
3nd tertile	0.67	(0.35 - 1.31)	0.87	(0.44 - 1.75)
Cabbages				
2nd tertile	1.17	(0.64 - 2.15)	1.92	(1.02 - 3.63)
3nd tertile	1.43	(0.77 - 2.66)	1.80	(0.94 - 3.47)
Mushrooms				
2nd tertile	1.38	(0.74 - 2.59)	1.70	(0.89 - 3.24)
3nd tertile	1.81	(0.98 - 3.34)	0.94	(0.46 - 1.94)
Onion and garlic				
2nd tertile	1.06	(0.57 - 1.95)	0.86	(0.46 - 1.61)
3nd tertile	0.57	(0.29 - 1.09)	0.54	(0.26 - 1.12)
Legumes				
2nd tertile	1.05	(0.59 - 1.84)	0.81	(0.43 - 1.52)
3nd tertile	0.92	(0.49 - 1.73)	0.60	(0.29 - 1.23)
Potatoes				
2nd tertile	1.45	(0.82 - 2.58)	1.29	(0.69 - 2.41)
3nd tertile	0.60	(0.32 - 1.13)	1.17	(0.65 - 2.12)
Fruits				
2nd tertile	1.23	(0.65 - 2.31)	0.95	(0.51 - 1.78)
3nd tertile	1.08	(0.52 - 2.24)	1.39	(0.65 - 2.95)
Citrus fruits				

2nd tertile	1.13	(0.65 - 1.99)	0.74	(0.37 - 1.46)
3rd tertile	1.26	(0.64 - 2.51)	0.94	(0.47 - 1.89)
All other fruits				
2nd tertile	0.78	(0.41 - 1.49)	1.01	(0.56 - 1.82)
3rd tertile	0.95	(0.47 - 1.92)	0.95	(0.46 - 1.97)
Dried fruit, nuts and seeds				
2nd tertile	1.53	(0.85 - 2.75)	1.18	(0.67 - 2.10)
3rd tertile	1.63	(0.86 - 3.10)	0.67	(0.37 - 1.22)
Sweets				
2nd tertile	1.12	(0.61 - 2.07)	1.06	(0.53 - 2.12)
3rd tertile	0.92	(0.45 - 1.85)	1.54	(0.80 - 2.98)
Chocolate, candy bars, etc.				
2nd tertile	0.95	(0.50 - 1.78)	1.65	(0.83 - 3.28)
3rd tertile	1.05	(0.59 - 1.89)	1.95	(1.07 - 3.58)
Sugar, honey, jam, confectionery				
2nd tertile	1.28	(0.68 - 2.38)	0.67	(0.36 - 1.24)
3rd tertile	1.40	(0.74 - 2.67)	0.66	(0.35 - 1.25)
Ice-cream				
2nd tertile	1.11	(0.62 - 1.98)	1.41	(0.76 - 2.59)
3rd tertile	0.78	(0.43 - 1.43)	1.24	(0.64 - 2.39)
Cakes, pies and pastries				
2nd tertile	1.26	(0.66 - 2.39)	2.44	(1.20 - 4.96)
3rd tertile	1.06	(0.56 - 1.99)	2.16	(1.08 - 4.29)
Biscuits, dry cakes				
2nd tertile	1.20	(0.66 - 2.16)	0.74	(0.40 - 1.38)
3rd tertile	0.81	(0.45 - 1.47)	1.00	(0.55 - 1.83)
Oils and fats				
2nd tertile	0.66	(0.37 - 1.18)	0.60	(0.31 - 1.15)
3rd tertile	0.56	(0.27 - 1.14)	0.66	(0.31 - 1.41)
Vegetables fats and non-olive oils				
2nd tertile	1.10	(0.59 - 2.06)	1.29	(0.60 - 2.77)
3rd tertile	0.85	(0.47 - 1.54)	0.57	(0.27 - 1.22)
Olive oil				
2nd tertile	0.47	(0.25 - 0.88)	0.78	(0.41 - 1.50)
3rd tertile	0.64	(0.33 - 1.25)	1.06	(0.54 - 2.08)
Butter and other animal fats				
2nd tertile	0.65	(0.34 - 1.24)	1.05	(0.55 - 2.00)
3rd tertile	1.05	(0.59 - 1.90)	1.04	(0.56 - 1.94)
Coffee				
2nd tertile	1.24	(0.71 - 2.16)	0.85	(0.48 - 1.52)
3rd tertile	1.43	(0.79 - 2.59)	0.67	(0.36 - 1.26)
Tea				
2nd tertile	1.10	(0.63 - 1.93)	1.84	(0.95 - 3.58)
3rd tertile	1.02	(0.55 - 1.88)	1.73	(0.93 - 3.23)
Red wine				
2nd tertile	0.79	(0.41 - 1.54)	2.06	(1.06 - 4.01)
3rd tertile	0.69	(0.37 - 1.28)	0.92	(0.46 - 1.86)
White wine				
2nd tertile	0.86	(0.45 - 1.66)	1.25	(0.64 - 2.45)
3rd tertile	0.69	(0.36 - 1.34)	1.63	(0.84 - 3.17)
Aperitif wines and beers				
2nd tertile	0.79	(0.41 - 1.54)	1.46	(0.71 - 3.01)
3rd tertile	0.89	(0.47 - 1.68)	1.17	(0.58 - 2.35)
Spirits and liqueurs				
2nd tertile	0.97	(0.49 - 1.92)	0.63	(0.29 - 1.37)
3rd tertile	0.97	(0.44 - 2.14)	1.05	(0.52 - 2.10)
Fruit juices				
2nd tertile	1.46	(0.72 - 2.97)	2.11	(1.06 - 4.20)
3rd tertile	1.71	(0.88 - 3.33)	1.18	(0.58 - 2.40)
Soft drinks				
2nd tertile	0.92	(0.49 - 1.74)	0.73	(0.36 - 1.48)
3rd tertile	0.74	(0.38 - 1.41)	1.24	(0.59 - 2.59)

[a]Adjusted for phototype, sunburn history, education, body mass index, non-alcohol energy, vitamin C and vitamin D intake, Greek Mediterranean index and glycemic index.
